# Widespread polyandry in an invasive beetle species (*Aethina tumida*)

**DOI:** 10.1038/s41598-026-52114-5

**Published:** 2026-05-06

**Authors:** Aura K. Palonen, Alexis L. Beaurepaire, Robine Schoch, Érica Weinstein Teixeira, Geoffrey R. Williams, Jay D. Evans, Francisco Posada-Florez, Christian W. W. Pirk, Akinwande K. Lawrence, Adewale A. Sorungbe, Giovanni Federico, Giovanni Formato, Robert Spooner-Hart, Clarissa M. House, Peter Neumann, Anna Papach

**Affiliations:** 1 Institute of Bee Health, University of Bern, Bern, Switzerland; 2 Swiss Bee Research Centre, Agroscope, Bern, Switzerland; 3 Instituto Biológico, Agência Paulista de Tecnologia dos Agronegócios, São Paulo, Brazil; 4 Department of Entomology & Plant Pathology, Auburn University, Auburn, USA; 5 U.S. Department of Agriculture -Agricultural Research Service, Bee Research Laboratory, Beltsville, USA; 6 Social Insects Research Group, University of Pretoria, Pretoria, South Africa; 7 Department of Biology, Federal University of Technology, Akure, Nigeria; 8 Laboratorio di Patologia Apistica, Istituto Zooprofilattico Sperimentale del Mezzogiorno, Reggio Calabria, Italy; 9 Istituto Zooprofilattico Sperimentale del Lazio e della Toscana “M. Aleandri”, Rome, Italy; 10 Hawkesbury Institute for the Environment, Western Sydney University, Richmond, Australia; 11 School of Science, Western Sydney University, Penrith, Australia

**Keywords:** Animal behaviour, Population genetics, Invasive species, Polyandry, Mating systems, Ecology, Ecology, Evolution, Genetics, Zoology

## Abstract

Biological invasions may induce adaptive shifts in traits that increase individual reproductive success, thereby accelerating population growth and amplifying ecological impacts. One such trait is polyandry, i.e. multiple mating by females, which may vary between endemic and invasive populations due to variation in demographic and environmental factors that alter the costs and benefits of multiple mating. Despite polyandry being widespread across taxa, comparative studies among multiple endemic and invasive populations of invasive species remain scarce. Here, we estimated the prevalence of polyandry in two endemic and five invasive populations of small hive beetle (*Aethina tumida*), a globally invasive parasite affecting bee health. Using DNA microsatellite genotyping of field-sampled females and their offspring to estimate mating frequencies, we show that polyandry varies in SHB populations and females mate with 1–13 males. However, the polyandry levels were not significantly different across the endemic and invasive populations compared. These results suggest that polyandry in SHB may be a stable reproductive trait rather than a plastic response to invasion-related factors. Our work supports further research on how multiple mating may improve both individual reproductive success and population viability in the context of biological invasions.

**Supplementary Information:**

The online version contains supplementary material available at 10.1038/s41598-026-52114-5.

## Introduction

The reproductive strategy of an invasive species commonly impacts the success of its establishment and expansion in novel ranges^1^. Additionally, selection pressures in novel ranges may drive adaptive shifts in reproductive traits in invasive populations, which may accelerate population growth and therefore further increase ecological impacts^2^. Consequently, a better understanding of mating systems of invasive species can help produce accurate predictions of invasion impact and support the development of management strategies^3,4^. However, large-scale studies comparing reproductive traits across endemic and invasive populations of a species remain scarce.

Polyandry, i.e. multiple mating by females, commonly varies across and within animal populations in both frequency and degree^5^. Polyandry may benefit invasive species by reducing inbreeding and maintaining genetic diversity in small or founding populations, thereby potentially facilitating successful invasions^6–8^. Offspring of polyandrous females tend to be more genetically diverse compared to offspring of monandrous females^9^. This may translate to higher survival in unpredictable environmental conditions or small demes, such as invasive populations^9,10^. Additionally, polyandry may ensure a female’s ability to reproduce in case a proportion of males in the population are genetically incompatible or infertile^11–13^. Indeed, multiple mating often increases female lifetime reproductive success across taxa, evident both in the occurrence of polyandry and in variation in mating frequency^14–16^.

The costs and benefits of polyandry are often context-dependent^17^. For example, polyandry levels may be driven by demographic factors such as population density^18,19^ and operational sex ratio^20^ since they affect the rate of mate encounters and mate-finding costs. Consequently, the low population densities typically associated with recent invasions may result in lower polyandry levels due to lower rate of mate encounters and higher costs of mate-finding^21,22^. Despite the widespread occurrence of polyandry across taxa^5,14^ and its potential role in successful invasions^6^, surprisingly little attention has been paid to it in this context.

The small hive beetle (*Aethina tumida*, SHB) is an invasive parasite that often infests colonies of the western honey bee, *Apis mellifera*^23,24^, but is also associated with other species of social bees^24^. Adult SHBs mate and reproduce inside their host’s nests, and the hatching larvae feed on hive resources such as pollen, honey and bee brood until they reach the post-feeding stage and leave the nests to pupate in nearby soil^23,24^. Adult SHB emerging from the soil will then disperse to locate a host colony and may fly distances up to 12 km[^25^]. Small hive beetle is endemic to sub-Saharan Africa^23^ but has spread to all continents except for Antarctica within the last 30 years^24,26–28^. In its endemic range, SHBs usually do not cause considerable damage to their hosts^29,30^, but in the invasive ranges they can destroy entire honey bee colonies by mass reproducing, which leads to thousands of SHB larvae taking over the nest^24,31^. The more common mass reproduction in the invasive populations of SHB has been suggested to be driven by quantitative differences in a range of behaviours between native and novel hosts, for example less frequent aggression towards SHB by novel honey bee hosts^24,32^. Both male and female SHB are known to mate frequently^33^, and females were shown to be polyandrous in one invasive population^34^. Female SHB can also store sperm in the spermatheca for later fertilization^35^. However, it remains unclear whether polyandry is a common mating strategy in this species and whether its reproductive biology varies between endemic and invasive populations.

In this study we estimated and compared levels of polyandry in endemic and invasive SHB populations using DNA parentage analysis of field-caught females and their offspring. Since SHB populations display striking differences in population density, for example showing higher densities in the endemic range compared to the invasive population in Italy^36^, and because population density can influence polyandry by affecting the rate at which females encounter potential mates^18,19^, we expected to find significant differences in polyandry between populations.

## Results

Out of the 2,243 SHB samples, a total of 1,867 offspring from 75 mothers were successfully genotyped at the ten microsatellite loci. Because the markers were located on the X chromosome^37^, offspring assumed to be haploid males were removed (see Methods section below), leaving 889 individuals for further analyses (Table 1). To determine whether any female offspring that were homozygous across all loci (and thus indistinguishable from haploid male offspring) were removed from the data, the proportion of mothers that were homozygous across all loci at each population was checked. The proportion was 0/12 in RSA and Nigeria, 0/9 Italy, 2/15 in Australia and 3/12 in both Alabama and Maryland in the USA.

Due to the low remaining sample size of offspring per family after removing putative haploid males (Median: 2.5, range: 2–6, Supplementary Table S1), the data from Brazil were excluded from further comparisons of polyandry between populations. Yet, polyandry levels estimated with allele counts confirmed that SHB is polyandrous in Brazil, with at least two of the eight females multiple mated (Supplementary Table S1). Basic population genetic metrics were still calculated for Brazil and are presented for comparison (Table 1).

**Table 1:** Population genetics analysis of seven populations of the small hive beetle (*Aethina tumida*). The first row of each population is based on the full dataset, and the second row is based on the dataset where putative haploid males were removed. For each population the number of successfully genotyped samples (field-captured females and their laboratory-reared offspring) and polymorphic loci (out of 10) are shown, along with non-detection error (NDE), mean and standard deviation of number of alleles (Na), observed (Ho) and expected heterozygosity (He), as well as the χ2 and p-values of testing for Hardy–Weinberg equilibrium (***, *p* < 0.001).

Population	*N* females	*N* polym. loci	*N* offspring	NDE (%)	Na Mean (SD)	Ho Mean (SD)	He Mean (SD)	χ2 value	*p*
RSA	12	10	276	0.1	4.5 (2.6)	0.241 (0.144)	0.447 (0.262)	230.3	***
			154	0.1	4.5 (0.8)	0.425 (0.08)	0.462 (0.083)	116.9	***
Nigeria	12	9	336	0	6.2 (2.7)	0.291 (0.127)	0.581 (0.219)	207.2	***
			149	0	6.2 (0.9)	0.612 (0.091)	0.585 (0.077)	139.7	***
USA Alabama	12	5	316	1.2	3.8 (1.5)	0.314 (0.077)	0.563 (0.167)	115.1	***
			136	0.9	3.8 (0.7)	0.687 (0.084)	0.593 (0.067)	61.9	***
USA Maryland	12	7	291	0.5	3.7 (2.1)	0.239 (0.147)	0.475 (0.273)	161.2	***
			124	0.4	3.7 (0.8)	0.528 (0.122)	0.478 (0.104)	57.3	***
Australia	11	7	259	0.3	3.7 (1.7)	0.314 (0.077)	0.546 (0.124)	161.2	***
			148	0.2	4.3 (0.8)	0.535 (0.050)	0.571 (0.047)	75.9	***
Italy	8	9	205	0.1	3 (0.7)	0.244 (0.084)	0.531 (0.169)	207.2	***
			83	0.1	3 (0.2)	0.568 (0.065)	0.521 (0.061)	56.6	***
Brazil	8	5	207	NA	2.3 (0.5)	0.105 (0.163)	0.117 (0.165)	32.2	***
			20	NA	2.4 (0.3)	0.317 (0.112)	0.252 (0.078)	6.2	0.623

The software Genepop v4.7[^38,39^] was used to test the conformity of all populations to Hardy-Weinberg equilibrium and the linkage disequilibrium between the microsatellite loci. All populations except for Brazil deviated from Hardy-Weinberg equilibrium (Table 1). All but two of the tested pairs of loci were at linkage disequilibrium (Supplementary Table S2). The observed and expected heterozygosity calculated with software GenAlEX v6.5^40,41^ ranged between 0.317 and 0.687 and 0.252–0.593 per population, respectively (Table 1). Heterozygosity estimates showed no consistent pattern, although values were lower in Brazil. This may partly reflect the exclusion of individuals homozygous across all loci (potentially both true homozygous females and haploid males), which may have disproportionately affected invasive populations such as Alabama and Maryland (USA) where 25% of the field-caught females were homozygous at all loci.

Genetic diversity was generally higher in the endemic populations of RSA and Nigeria compared to the invasive ones. The native SHB populations showed both a high number of polymorphic loci (RSA: 10 out of 10, Nigeria: 9 out of 10; Table 1) and high mean number of alleles per locus (RSA: 4.7, Nigeria: 6.2; Table 1). Genetic diversity metrics also varied among the invasive populations. In the invasive population of Italy, 9 loci were polymorphic, but the allelic diversity was lower overall, with only 3 alleles per locus on average and no strong differences in allele numbers across loci (Table 1). In contrast, the invasive population in Alabama (USA) only had 5 polymorphic loci, but the mean number of alleles per locus was 3.8 (Table 1). The invasive population in Brazil showed the lowest diversity across both measures, with 5 polymorphic loci and a mean of 2.4 alleles per locus (Table 1).

To determine whether the remaining sample sizes sufficiently captured the allelic richness in the populations, a rarefaction analysis was done using the software ADZE^42^ based on the genotype data of all field-caught females. The rarefaction curves in RSA, Nigeria, Alabama (USA), Maryland (USA) and Australia reached a plateau at approximately 25 individuals (Supplementary Fig. S1a‒e). In Italy the curve plateaued at around 15 individuals (Supplementary Fig. S1f). Our sample sizes of 4–11 females per population used in the final polyandry analyses fell slightly below this plateau, capturing between 61 and 82% of the allelic richness (Supplementary Fig. S1a‒f).

Polyandry was estimated using two methods: COLONY v2.0.7.1. software^43^ and paternal haplotype counts (see Methods below). To estimate the minimum number of offspring per brood for robust polyandry estimates, a rarefaction analysis was done for both the data obtained from COLONY software and the paternal haplotypes data. The number of sires detected increased with the number of offspring sampled and began to plateau at approximately 14‒16 offspring for both polyandry estimates (Supplementary Fig. S2a‒g; Supplementary Fig. S3a‒g). The minimum number of offspring per brood was thus determined as 10. While this threshold was lower than the plateau, it captured approximately 60‒100% of the sires detected with the full sample size and allowed more broods to be retained for the polyandry analysis (Supplementary Fig. S2a‒g; Supplementary Fig. S3a‒g). In addition, non-sampling errors were calculated^44^ for each brood to estimate how many sires may have remained undetected by the COLONY software due to incomplete sampling of offspring (i.e., limited sample size), and they ranged between 0 and 3.7 (median: 0.8) (Supplementary Table S3).

**Fig. 1:**
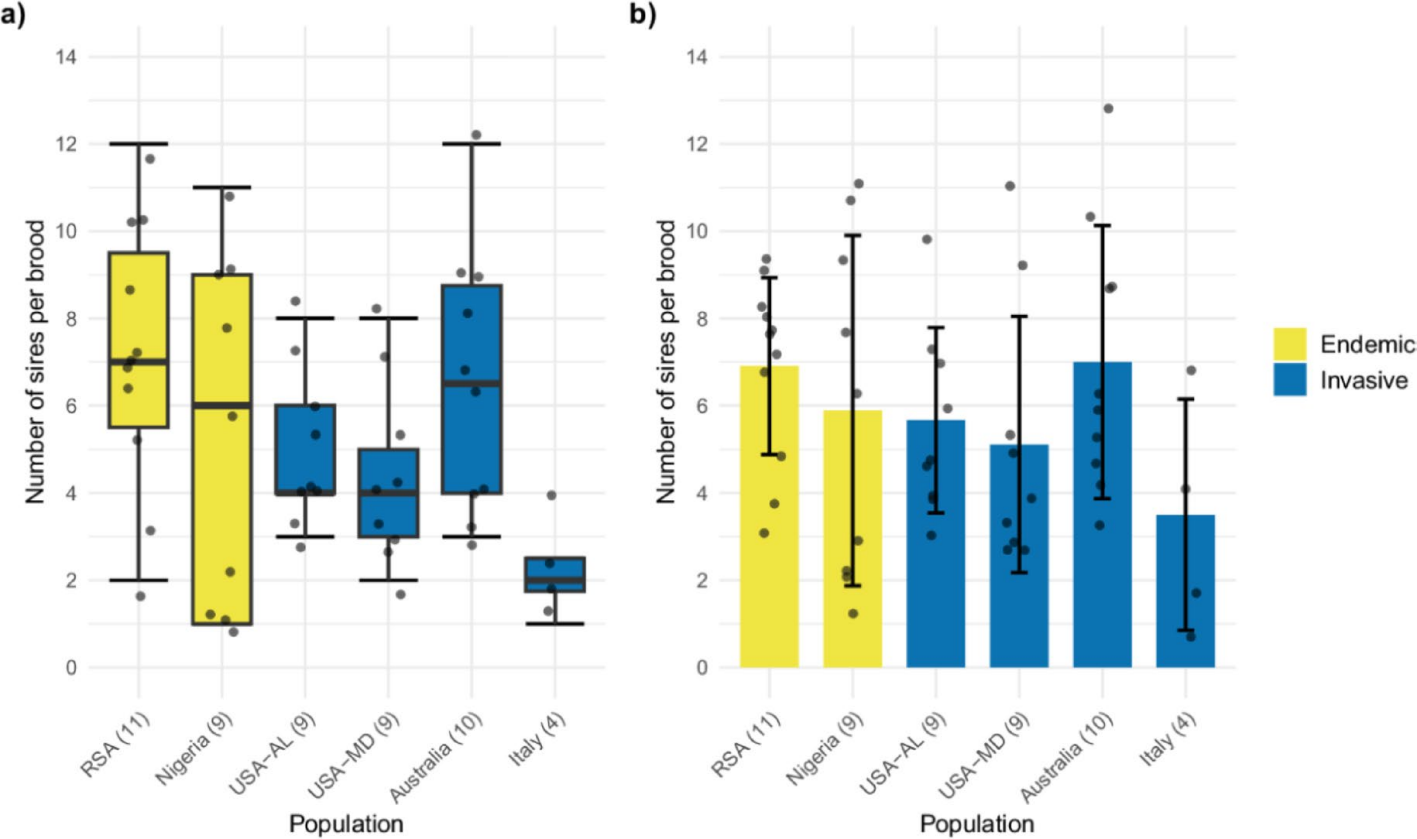
Polyandry across six populations of the small hive beetle (SHB, *Aethina tumida*). The sample size of each population is indicated in brackets. There were no significant differences in level of polyandry between the populations (Kruskal-Wallis test/ANOVA all *p* > 0.05). (**a**) Estimates from COLONY software. Medians (centre lines), interquartile ranges (boxes), overall ranges excluding outliers (whiskers) and individual data points (dots) are shown. (**b**) Estimates from haplotype counting. Means (top of the bars), standard deviations (whiskers) and individual data points (dots) are shown.

After excluding the Brazil samples and the broods with less than 10 offspring, the final sample size for the polyandry analyses was 700 offspring from 52 mothers (Supplementary Table S3). Broods typically showed more than one paternal allele (range 1–7; Supplementary Table S3). The polyandry estimates from COLONY software ranged between 1 and 12 (median: 5) (Fig. 1a). The polyandry estimates from paternal haplotype counting ranged between 1 and 13 (mean: 6) (Fig. 1b). Sire number estimates derived from paternal haplotypes closely matched those obtained using COLONY (linear regression R^2^ = 0.71, *p* < 0.001). The Kruskal-Wallis tests showed no statistically significant differences (all p-values > 0.05) in polyandry between the populations using the software estimates (χ2 = 10.23, df = 5, *p* = 0.069) (Fig. 1a). Similarly, no significant differences were found in the paternal haplotype counts with an ANOVA (F = 1.253, df = 5, *p* = 0.301). The number of sires per brood inferred using COLONY software was highest in RSA (median: 7, range: 2–12) and lowest in Italy (median: 2, range: 1–4). Using haplotype counts, the number of sires per brood was highest in Australia (mean: 7, range: 3–13) and lowest in Italy (mean: 3.5, range: 1–7). Non-sampling errors of the polyandry estimates inferred with COLONY were higher in RSA (range: 0‒2.9), Nigeria (range: 0‒3.1) and Australia (range: 0.1‒3.4), and lower in Italy and the USA populations (Supplementary Table S3). Non-detection errors that estimated the likelihood of sires remaining undetected, despite being sampled, due to multiple sires sharing indistinguishable genotypes were low and ranged between 0.1% and 0.9% per population, with a median of 0.15% (Table 1).

## Discussion

Our results show for the first time that polyandry is widespread in SHB as females mated multiple times in the two endemic and five invasive populations studied here. The level of polyandry did not significantly differ between the populations, which suggests that female multiple mating is a ubiquitous reproductive trait in the SHB.

Both the number of polymorphic microsatellite loci and the mean number of alleles per locus were generally higher in the endemic populations in RSA and Nigeria than in the invasive populations in the USA (Alabama and Maryland), Australia, Italy and Brazil. This difference in allelic richness and marker polymorphism is a likely consequence of genetic bottlenecks following invasions^1^. Marker polymorphism and allelic richness were lowest in Brazil, which may be due to our limited sample size and/or the population being a secondary invasion originating from the USA^45^. Despite the differences in marker polymorphism and allelic richness, the rarefaction analysis showed that in all populations where we estimated polyandry (i.e., excluding Brazil) the sample size captured a substantial share of the genetic diversity in the populations. The relatively low allelic richness across the populations studied may be due to the lower genetic diversity usually observed in sex chromosomes^46^, as supported by a previous study using autosomal microsatellite markers in the SHB finding higher allelic richness than the current one^47^.

All populations except for Brazil showed significant deviations from Hardy-Weinberg equilibrium (HWE), which is unlikely to have affected the polyandry estimates as the COLONY software used to estimate paternity is able to account for such deviations. All but two pairs of loci were in linkage disequilibrium, most likely due to the markers used being located on the X chromosome^37^. While a previous study shows that moderately linked markers only slightly impact the accuracy of parentage inference^48^, our tightly linked markers may have reduced the accuracy of the software. However, the impact seems to be small, since the polyandry estimates obtained from paternal haplotype counting closely matched those obtained from the software. Still, future studies on polyandry in this species would benefit from incorporating autosomal markers better suited to paternity reconstruction.

Several factors could have caused uncertainty in the polyandry estimates, and the magnitude of the effects likely differs across populations. Because the markers were on the X chromosome, we removed all offspring that displayed a unique allele at every locus (i.e. possible haploid males). This likely also included some diploid homozygous female offspring. Based on the maternal genotypes, the probability of a female being homozygous across all loci was highest in the populations in USA (Alabama and Maryland) and Australia, suggesting that we excluded at least some paternal alleles from the data and therefore underestimated the number of sires in these populations. In contrast, no mothers were homozygous across all loci in Italy and the endemic populations in RSA and Nigeria, suggesting that this filtering may not have impacted polyandry estimates in these populations. Furthermore, the presence of null alleles may affect the estimates of multiple paternity, but we were unable to assess this effect due to the deviations from HWE^49^. Accounting for null alleles would result in a higher number of contributing males, which further suggests that our estimates of polyandry are likely conservative. Finally, polyandry might also be underestimated due to insufficient sampling of offspring, especially in RSA, Nigeria and Australia which tended to have higher non-sampling errors (NSE) and lower percentages of detected sires in the rarefaction analyses. Taken together, these patterns suggest that our polyandry estimates are likely conservative, with underestimation arising from insufficient sampling in RSA, Nigeria and Australia and from the removal of some paternal alleles in the USA populations. Nevertheless, the consistency of our results across both estimates of polyandry suggests that the absence of differences between the endemic and invasive populations studied here remains valid.

Out of 52 studied females only four (three from the Nigeria population and one from the Italy population) were not multiply mated, showing that polyandry is widespread in the SHB, with all studied populations showing multiple paternity. The variation in the frequency of polyandry may be explained by factors such as age differences in field-sampled females, i.e. single mated females were younger^50^. The absence of significant differences in polyandry between populations, even when comparing the endemic high density populations in RSA and Nigeria and the invasive extremely low density population in Italy^36^, suggests that population density is not a major driver of polyandry in the SHB. It seems that even in populations with very low population densities, e.g. Italy, SHB females encounter enough suitable males to mate multiply. This may be explained by the tendency of SHB to move frequently within apiaries and their ability to disperse over long distances^25,51^. SHB are also documented to cluster in specific host colonies, which probably increases mate encounter rates and mating frequency^33,51^. Furthermore, our results suggest that SHB females mate multiply even in new introductions where population densities are extremely low and that, consequently, polyandry may have facilitated the successful SHB invasions by mitigating the negative effects of inbreeding^8^. Future studies are however needed to elucidate the role of polyandry in facilitating invasion success of SHB.

Empirical evidence of potential adaptive shifts in polyandry mainly stems from the harlequin ladybird, *Harmonia axyridis*^52^, where polyandry has been reported to increase in invasive populations. However, similar levels of polyandry throughout the distribution of SHB suggest that polyandry is unlikely to be a plastic response to novel selection pressures in invasive ranges in this species. Rather, polyandry appears to be a ubiquitous reproductive trait that can enhance both individual reproductive success and population survival. For example, in the invasive mosquitofish (*Gambusia holbrooki*) polyandry levels are similar across endemic and invasive ranges, but multiple mating is suggested to be favoured since it is likely to provide benefits such as reducing inbreeding, increasing the effective population size, and boosting both male and female reproductive success^53^. This may also be the case in the SHB. While the specific benefits of polyandry for SHB females remain unclear, they have been hypothesized to mate multiply to ensure that the spermatheca is filled should the opportunity for reproduction arise^34^. Given that the opportunities for SHB reproduction are brief and unpredictable^24,32^, females may gain an advantage by mating quickly to ensure they are able to reproduce. Additionally, since SHB larvae may encounter variable environmental conditions when leaving the host nest to pupate, polyandry may act as a bet-hedging strategy to maximize offspring survival in unpredictable environmental conditions^9^. Other possible benefits of polyandry in the SHB include the opportunity to improve offspring viability by biasing paternity towards the most suitable male (i.e. cryptic female choice^9,54,55^. While polyandry may occur simply because the costs of not mating at all are high^21^, our observation of ubiquitous polyandry across populations indicates it may directly contribute to the ecological success of this and possibly other invasive species, highlighting the need for further research on the role of mating systems in facilitating successful invasions.

Our study provides one of the most comprehensive comparisons of polyandry across the distribution of an invasive species, offering new insights into the stability of mating strategies across endemic and invasive populations. We show that polyandry is ubiquitous in both endemic and invasive SHB populations, with no significant differences in its levels among populations despite differences in infestations, climate and hosts^36^. To our knowledge, this represents the first evidence of large-scale polyandry in a sap beetle (Coleoptera: Nitidulidae). Our findings suggest that polyandry is unlikely to be driven by population density or novel selection pressures in invasive regions. Instead, its widespread occurrence may reflect material or genetic benefits to females. Our findings highlight the need for future research on how polyandry may contribute to the establishment and spread of SHB and other invasive species.

## Methods

### Beetle rearing

Polyandry was estimated in two endemic (South Africa, hereafter RSA (Gauteng) and Nigeria (Osun and Ondo) and five invasive (Australia (New South Wales), Brazil (Rio de Janeiro), Italy (Reggio Calabria) and the USA (Alabama and Maryland) SHB populations. At each population, SHBs were collected from 9 to 15 honey bee colonies located in two or three different apiaries. The SHBs were sexed^56^ and a randomly chosen female from each screened honey bee colony was used to start standard laboratory rearing^57^. To do so, the field-collected SHB females were placed individually in jars with punctured lids, provided 1:2 honey-pollen paste *ad libitum*, oviposition sites (two microscope slides separated by two square cover slips at the ends and taped together) and incubated at 25 °C 80% relative humidity (RH) following standard methods^57^. The jars were checked daily to ensure that hatching larvae had sufficient food until they reached the post-feeding stage (i.e., wandering larvae^23^. Wandering larvae from 81 mothers (*N* = 20–40 each) were collected and placed to pupate (larvae from the same mother were placed together) in jars 75% filled with ~ 10% moisture by mass, autoclaved commercially available all-purpose sand (except for RSA, where autoclaved local soil was used)^57^. The jars were incubated at 25 °C 80% RH in constant darkness until adult SHBs emerged (Neumann et al. 2013). The field-caught females and their adult offspring were freeze-killed at -20 °C and stored in 75% EtOH for at least 48 h before transport to the Institute of Bee Health, University of Bern, Switzerland for genotyping.

### Genotyping

A total of 81 field-collected females and 15–36 offspring/female were used. DNA of all offspring and 21 of the mothers was extracted with a standard 5% Chelex protocol^58^ using two legs per beetle. For the rest of the mothers (*N* = 60), DNA was extracted with the NucleoSpin^®^ Tissue kit (Macherey-Nagel). The individuals were placed in 2 mL tubes with TN buffer (100 µl) and a metal bead and then homogenized for one to three minutes at a frequency of 25/s. A total of 50 µl of the homogenized sample was then used to extract DNA according to the manufacturer’s instructions. The DNA quality of the samples extracted with the kit was estimated using a QuickDropTM spectrophotometer (Molecular Devices, San Jose, California, USA) and diluted in case it was higher than 100 ng/µL to ensure successful replication of DNA during the PCR.

The samples (*N* = 2,243) were genotyped using 10 DNA microsatellite markers (Supplementary Table S4), 5 of which have been previously published^34^. The primers were developed directly from the SHB genome retrieved from NCBI (accession number: NW_017852934.1). Primers were developed using the Primer3Plus software^59^. The PCR product sizes were selected in order to allow the combination fluorophore dyes in multiplex PCR amplifications.

Genotyping of the samples was done using the KAPA2G Fast Multiplex PCR Kit (Kapa Biosystems) and 1 µl of DNA: 5 min 95 °C, followed by 35 cycles of 30 s at 95 °C, 30 s at 60 °C and 30 s at 72 °C and a final elongation of 5 min at 72 °C. The thermocyclers used were QIAamplifier 96 (Qiagen^®^), Biometra Tprofessional (Analytik Jena^®^) and Biometra Tadvanced (Analytik Jena^®^). PCR products were diluted and sent to Microsynth AG (Balgach, Switzerland) to be run on a 3730xl DNA Analyzer (Applied Biosystems^®^). Genotypes of the individuals were determined manually using PeakScanner software v1.0 (Applied Biosystems^®^). In case an individual had missing data at more than half of the polymorphic loci after repeating the PCR twice, the individual was removed from the dataset. When a female was successfully genotyped but her offspring were not, the female was still included in analyses based on maternal genotypes.

### Genetic analyses

After genotyping all samples, a corrigendum to the original publication^34^ was released indicating that the microsatellite markers used are all located on the X-chromosome^37^. Since the offspring were not sexed prior to DNA extraction, and sample deterioration prevented subsequent sex determination, it was not possible to distinguish between male offspring (i.e., effectively haploid since they only have one X-chromosome) and diploid female offspring that were homozygous across all loci. To avoid misinterpreting the single allele per locus in male offspring as a unique paternal allele and consequently overestimating polyandry levels, a conservative approach was chosen: all individuals that appeared homozygous at all loci (i.e., possible males) were excluded from the data.

For each population, observed and expected heterozygosity levels were calculated with GenAlEX v6.5^40,41^ and conformity to Hardy–Weinberg equilibrium was tested with Genepop v4.7. web version^38,39^. Additionally, data from all populations were pooled to estimate linkage disequilibrium between each pair of loci with Genepop v 4.7. web version^38,39^. To estimate marker resolution, a non-detection error was calculated for each population^44^. A rarefaction analysis was done for each population using the software ADZE^42^, to estimate whether the sample sizes used sufficiently captured the genetic diversity in each population.

Three approaches were used to estimate polyandry levels. First, the number of sires per brood was estimated by direct allele counting, defined as the number of non-maternal alleles at the locus with greatest allelic diversity^44^. Second, the software COLONY v2.0.7.1^43^ was used to estimate the number of sires in each brood. The following settings were chosen for the COLONY runs: mating system with both male and female polygamy; inbreeding without clone; haplodiploid dioecious species; medium length run with full likelihood analysis method; no sib ship prior. Due to the markers being on the same chromosome and therefore linked (Supplementary Table S2), the accuracy of the COLONY software may be lower^48^. We therefore also estimated polyandry using paternal haplotype counts, assuming complete linkage among markers. For each offspring, paternal alleles were identified by removing the maternal allele from the genotype at each locus. The remaining paternal alleles were combined across loci to reconstruct paternal haplotypes, and the number of unique haplotypes per brood was used as the minimum number of sires. In case the mother and offspring shared the same heterozygous genotype, the paternal allele was marked as missing data. Haplotypes differing only at a locus with missing data were not considered different. To evaluate whether the polyandry estimates obtained from haplotype counts matched those from COLONY software, the data were fitted to a linear regression.

To determine the minimum number of offspring per brood for robust polyandry estimates, we performed a resampling analysis to evaluate how sire number estimates varied with sample size^60^. This analysis was done separately for COLONY software outputs and paternal haplotype counts. For COLONY, the software was first run using a subset of two offspring, then adding two randomly sampled offspring at a time and repeating the analysis until the full sample size of the brood. This was repeated for the three largest broods per population. For the haplotype data, the number of unique paternal haplotypes was counted at each sample size, starting from one offspring, and the random sampling was repeated 100 times for each sample size using R v 4.5.1^61^. The number of unique paternal haplotypes was counted at each sample size starting from one, and this random sampling was repeated 100 times for each sample size. The minimum sample size of offspring per brood was then decided based on when the estimated sire numbers started to plateau in both analyses. Finally, using the COLONY output, a non-sampling error was calculated for each brood to estimate the number of sires that may have remained undetected due to possible insufficient sample size of offspring^44^.

### Statistical analyses

All statistical analyses were performed using R v 4.5.1^61^. Both polyandry estimates (number of sires per brood estimated with software and haplotype counts) were checked for normality with Shapiro-Wilk tests. The differences in polyandry levels between populations were compared with Kruskal-Wallis tests for the software data, and with an ANOVA for the haplotype data.

## Supplementary Information

Below is the link to the electronic supplementary material.

Supplementary Material 1

## Data Availability

The raw data of the study are available at figshare: 10.6084/m9.figshare.32020083.
