# Supplementary material for: Widespread polyandry in an invasive beetle species (Aethina tumida)
Source: Sci Rep. 2026 May 6;16:20946. doi: 10.1038/s41598-026-52114-5 (PMC13338151; doi:10.1038/s41598-026-52114-5)
Supplement: Supplementary file 1 — Supplementary Material 1 [file 41598_2026_52114_MOESM1_ESM.docx]

Scientific Reports

**Widespread polyandry in an invasive beetle species (*Aethina tumida*)**

Aura K. Palonen, Alexis L. Beaurepaire, Robine Schoch, Érica Weinstein Teixeira, Geoffrey R. Williams, Jay D. Evans, Francisco Posada-Florez, Christian W.W. Pirk, Akinwande K. Lawrence, Adewale A. Sorungbe, Giovanni Federico, Giovanni Formato, Robert Spooner-Hart,
Clarissa M. House, Peter Neumann and Anna Papach

Corresponding author: Aura Palonen; Institute of Bee Health, Vetsuisse Faculty, University of Bern, Bern, Switzerland; [aura.palonen@unibe.ch](mailto:aura.palonen@unibe.ch)

SUPPLEMENTARY MATERIAL


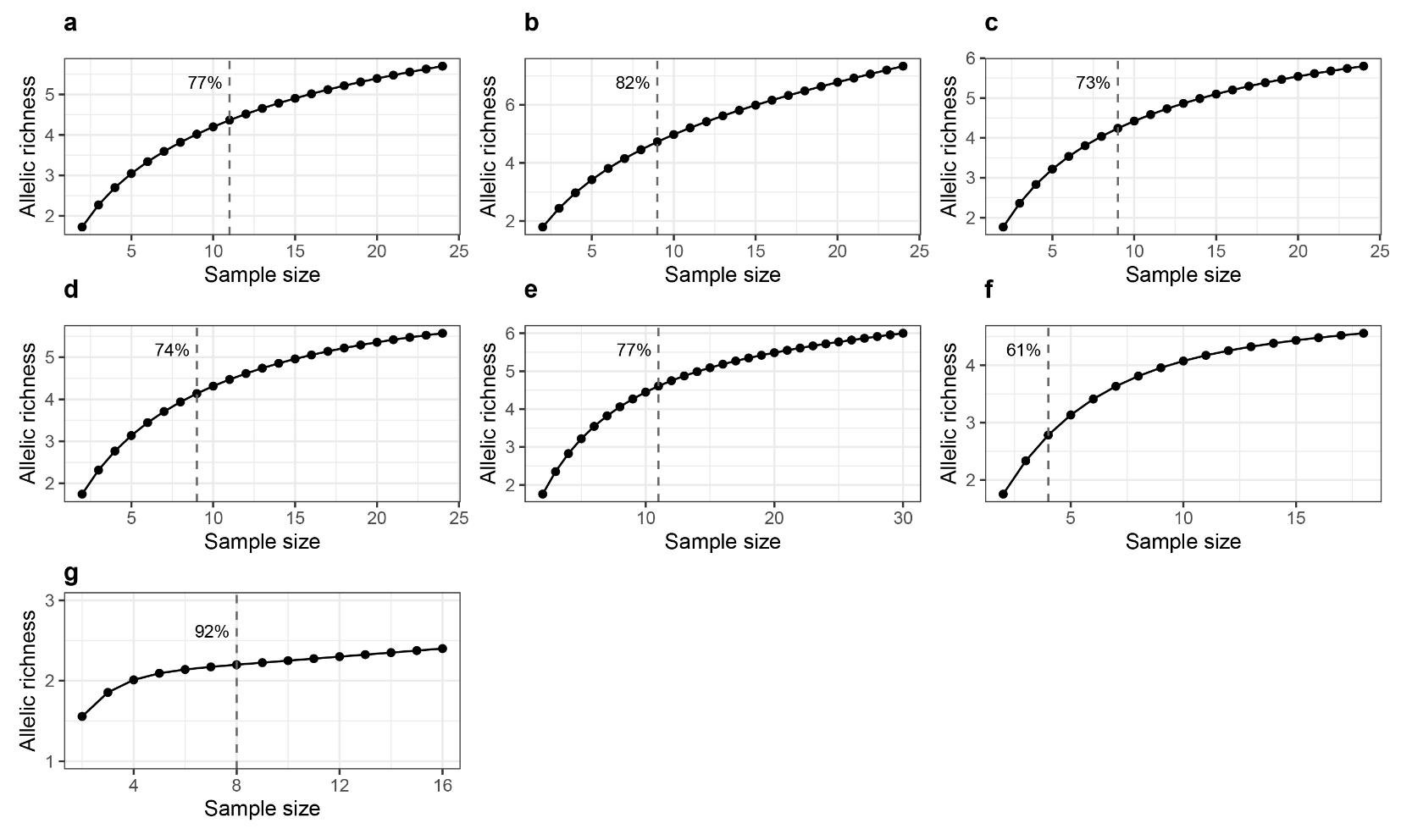


**Supplementary Figure S1a–f.** Rarefaction analysis on the allelic richness in six populations of small hive beetle (*Aethina tumida*). The rarefaction analysis was done with the software ADZE using genotype data of field-caught females. The dashed lines show the sample size used in further analyses and the percentage of allelic richness covered by this sample size is shown. **a)** RSA, **b)** Nigeria, **c)** Alabama (USA), **d)** Maryland (USA), **e)** Australia, **f)** Italy.

**
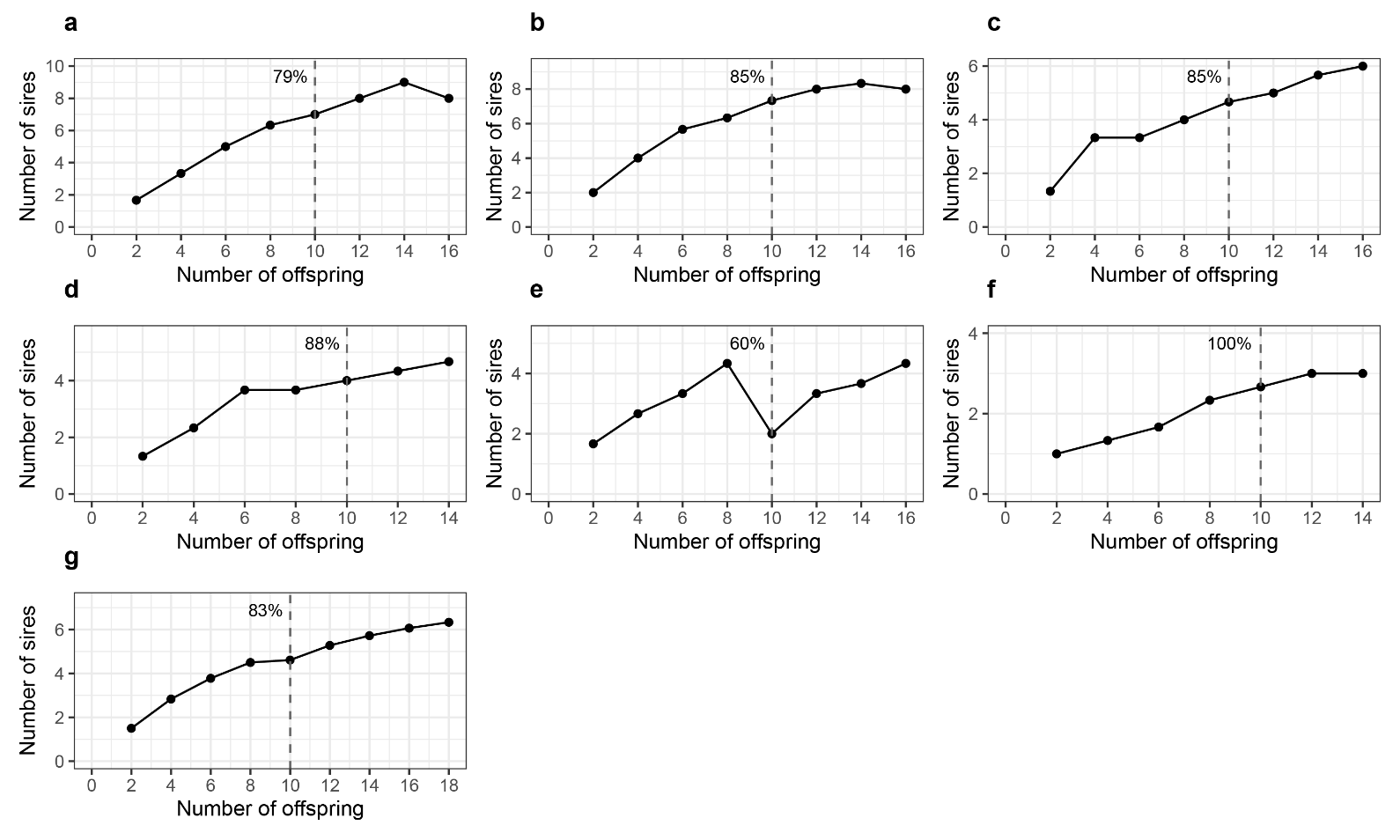
Supplementary Figure S2a–g.** The effect of sample size on the number of sires detected in seven populations of small hive beetle (*Aethina tumida*). The analysis was done by randomly sampling two offspring from each brood, running the data with COLONY software to infer the number of sires, and then adding two offspring at a time, rerunning COLONY for each sample size. This procedure was repeated for three broods per population, and the results were averaged across them. Dashed lines and percentages indicate the proportion of sires detected with a sample size of 10 offspring. **a)** RSA, **b)** Nigeria, **c)** Alabama (USA), **d)** Maryland (USA), **e)** Australia, **f)** Italy, **g)** The average across all six populations.

**
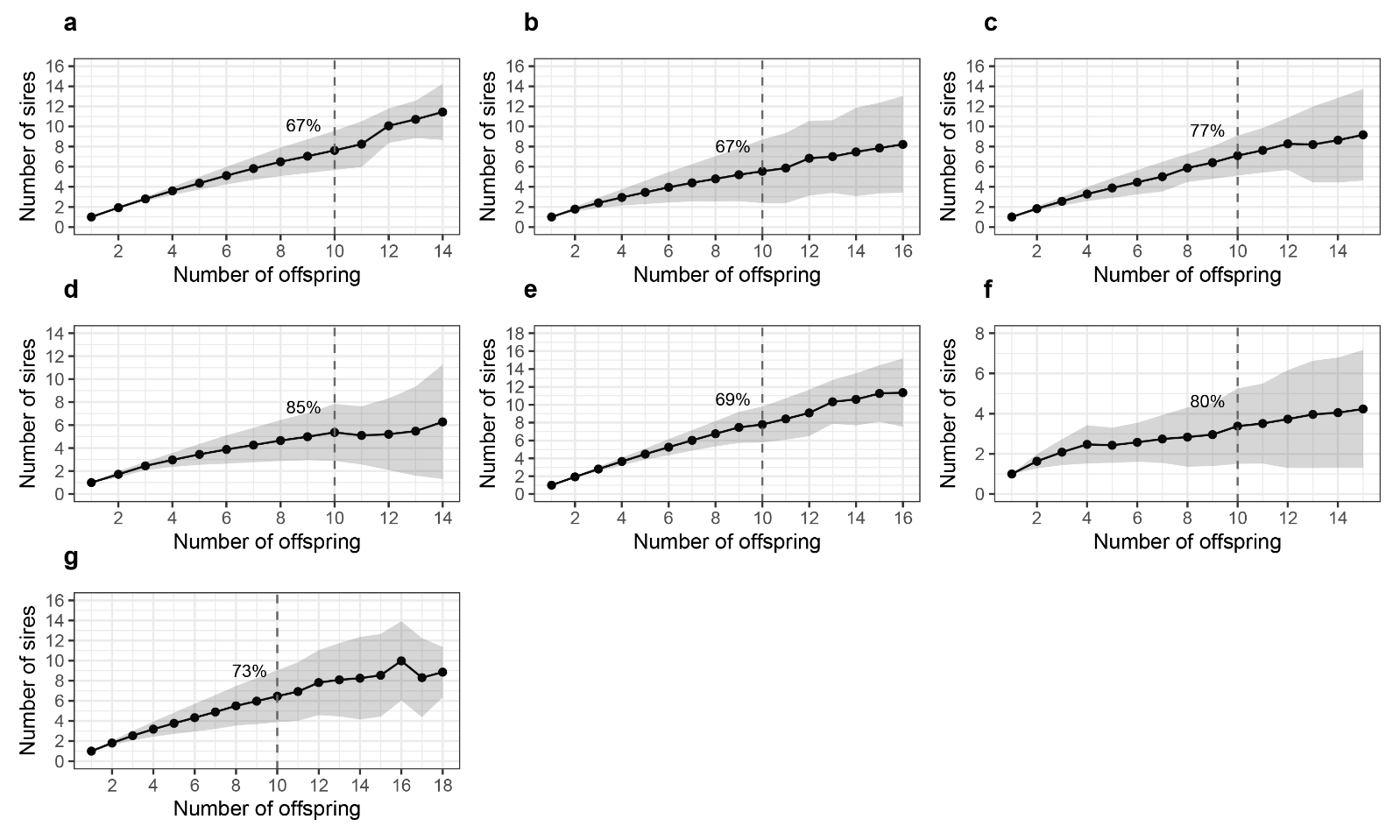
Supplementary Figure S3a–g.** The effect of sample size on the number of sires detected in seven populations of small hive beetle (*Aethina tumida*). The analysis was done by randomly sampling offspring and counting the number of unique paternal haplotypes (i.e., number of sires). Each sample size was repeated 100 times, and the number of sires was averaged across replicates. Dots indicate the mean, and shaded areas show the standard deviation. Dashed lines and percentages indicate the proportion of sires detected with a sample size of 10 offspring. **a)** RSA, **b)** Nigeria, **c)** Alabama (USA), **d)** Maryland (USA), **e)** Australia, **f)** Italy, **g)** The average across all six populations.

**Supplementary Table S1.** Paternity of 6 broods of small hive beetle (*Aethina tumida*) from the invasive Brazil population inferred from allele counts. Brood, apiary and colony IDs are shown, as well as the number of successfully genotyped offspring and number of nonmaternal alleles. Due to the small sample size, the Brazil samples were excluded from comparisons of polyandry between populations, and these data were only used to confirm multiple mating in this population.

| **Brood** | **Apiary** | **Colony** | **N offspring** | **N nonmaternal alleles** |
| --- | --- | --- | --- | --- |
| BR_1 | BR_A | BR_A1 | 2 | 1 |
| BR_2 | BR_A | BR_A1 | 6 | 0 |
| BR_3 | BR_A | BR_A2 | 3 | 1 |
| BR_4 | BR_B | BR_B1 | 4 | 2 |
| BR_5 | BR_B | BR_B2 | 2 | 2 |
| BR_6 | BR_B | BR_B3 | 2 | 1 |

**Supplementary Table S2.** Linkage disequilibrium between all DNA microsatellite loci used to estimate polyandry in the small hive beetle (*Aethina tumida*). Pairs of loci tested, χ2 values and p- values are shown. Locus pairs with a significant p-value (< 0.05) are in linkage disequilibrium.

| **Locus pair** | **χ2 value** | **p value** |
| --- | --- | --- |
| ATU16 & ATU18 | 101.629 | < 0.001 |
| ATU16 & ATU5A | 111.251 | < 0.001 |
| ATU18 & ATU5A | 191.309 | < 0.001 |
| ATU16 & ATU11 | 64.646 | < 0.001 |
| ATU18 & ATU11 | 117.687 | < 0.001 |
| ATU5A & ATU11 | 130.652 | < 0.001 |
| ATU16 & ATU9 | 63.770 | < 0.001 |
| ATU18 & ATU9 | 109.334 | < 0.001 |
| ATU5A & ATU9 | 87.962 | < 0.001 |
| ATU11 & ATU9 | 100.794 | < 0.001 |
| ATU16 & ATU8 | 21.113 | 0.007 |
| ATU18 & ATU8 | 127.255 | < 0.001 |
| ATU5A & ATU8 | 130.638 | < 0.001 |
| ATU11 & ATU8 | 40.138 | < 0.001 |
| ATU9 & ATU8 | 44.432 | < 0.001 |
| ATU16 & ATU17 | 112.640 | < 0.001 |
| ATU18 & ATU17 | 167.309 | < 0.001 |
| ATU5A & ATU17 | 191.309 | < 0.001 |
| ATU11 & ATU17 | 116.200 | < 0.001 |
| ATU9 & ATU17 | 67.256 | < 0.001 |
| ATU8 & ATU17 | 145.616 | < 0.001 |
| ATU16 & ATU14 | 81.794 | < 0.001 |
| ATU18 & ATU14 | 207.167 | < 0.001 |
| ATU5A & ATU14 | 191.309 | < 0.001 |
| ATU11 & ATU14 | 131.154 | < 0.001 |
| ATU9 & ATU14 | 100.419 | < 0.001 |
| ATU8 & ATU14 | 104.139 | < 0.001 |
| ATU17 & ATU14 | 179.765 | < 0.001 |
| ATU16 & ATU10 | 67.484 | < 0.001 |
| ATU18 & ATU10 | 129.309 | < 0.001 |
| ATU5A & ATU10 | 134.854 | < 0.001 |
| ATU11 & ATU10 | 130.286 | < 0.001 |
| ATU9 & ATU10 | 88.952 | < 0.001 |
| ATU8 & ATU10 | 79.743 | < 0.001 |
| ATU17 & ATU10 | 130.650 | < 0.001 |
| ATU14 & ATU10 | 128.253 | < 0.001 |
| ATU16 & ATU2 | 34.105 | < 0.001 |
| ATU18 & ATU2 | 72.160 | < 0.001 |
| ATU5A & ATU2 | 119.277 | < 0.001 |
| ATU11 & ATU2 | 91.575 | < 0.001 |
| ATU9 & ATU2 | 66.528 | < 0.001 |
| ATU8 & ATU2 | 13.665 | 0.091 |
| ATU17 & ATU2 | 95.295 | < 0.001 |
| ATU14 & ATU2 | 92.732 | < 0.001 |
| ATU10 & ATU2 | 84.530 | < 0.001 |

**Supplementary Table S3.** Paternity of 52 small hive beetle (*Aethina tumida*) broods inferred from the software COLONY and from paternal haplotype counts. Brood, population, apiary and colony IDs are shown, as well as the number of successfully genotyped offspring (N offspring total) and the number of offspring after removing putative males (N offspring filtered). The filtered dataset was used in paternity analyses. The number of paternal alleles is shown as a conservative baseline estimate of number of sires. The number of sires per brood inferred from the software and the corresponding non-sampling error estimates (NSE) are shown, as well as the number of paternal haplotypes per brood.

| **Brood** | **Population** | **Apiary** | **Colony** | **N offspring total** | **N offspring filtered** | **N paternal alleles** | **N sires COLONY** | **NSE** | **N paternal haplotypes** |
| --- | --- | --- | --- | --- | --- | --- | --- | --- | --- |
| RSA_1 | RSA | RSA_A | RSA_A1 | 25 | 13 | 3 | 7 | 1.5 | 8 |
| RSA_2 | RSA | RSA_A | RSA_A2 | 29 | 20 | 4 | 6 | 1.5 | 9 |
| RSA_3 | RSA | RSA_A | RSA_A3 | 28 | 11 | 2 | 2 | 0.2 | 3 |
| RSA_4 | RSA | RSA_A | RSA_A4 | 24 | 11 | 3 | 5 | 1.3 | 5 |
| RSA_5 | RSA | RSA_B | RSA_B1 | 23 | 11 | 3 | 3 | 0.0 | 4 |
| RSA_6 | RSA | RSA_B | RSA_B2 | 23 | 12 | 4 | 7 | 0.9 | 8 |
| RSA_7 | RSA | RSA_B | RSA_B3 | 24 | 13 | 5 | 10 | 2.5 | 8 |
| RSA_8 | RSA | RSA_C | RSA_C1 | 23 | 16 | 5 | 10 | 1.8 | 7 |
| RSA_9 | RSA | RSA_C | RSA_C2 | 25 | 13 | 4 | 7 | 1.3 | 9 |
| RSA_10 | RSA | RSA_C | RSA_C3 | 18 | 12 | 5 | 9 | 2.2 | 8 |
| RSA_11 | RSA | RSA_C | RSA_C4 | 17 | 14 | 4 | 12 | 2.9 | 7 |
| NG_1 | Nigeria | NG_A | NG_A1 | 28 | 17 | 1 | 1 | 0.0 | 1 |
| NG_2 | Nigeria | NG_A | NG_A2 | 30 | 14 | 1 | 1 | 0.0 | 2 |
| NG_3 | Nigeria | NG_B | NG_B1 | 30 | 13 | 4 | 6 | 1.1 | 6 |
| NG_4 | Nigeria | NG_B | NG_B2 | 30 | 12 | 2 | 2 | 0.2 | 3 |
| NG_5 | Nigeria | NG_B | NG_B3 | 27 | 11 | 1 | 1 | 0.0 | 2 |
| NG_6 | Nigeria | NG_C | NG_C1 | 30 | 16 | 7 | 8 | 1.4 | 11 |
| NG_7 | Nigeria | NG_C | NG_C1 | 30 | 19 | 4 | 9 | 2.4 | 11 |
| NG_8 | Nigeria | NG_C | NG_C2 | 24 | 12 | 5 | 11 | 3.1 | 8 |
| NG_9 | Nigeria | NG_C | NG_C2 | 29 | 14 | 5 | 9 | 2.0 | 9 |
| AL_1 | USA-Alabama | AL_A | AL_A1 | 26 | 18 | 3 | 4 | 0.2 | 4 |
| AL_2 | USA-Alabama | AL_A | AL_A2 | 25 | 12 | 3 | 4 | 0.2 | 7 |
| AL_3 | USA-Alabama | AL_A | AL_A3 | 27 | 12 | 2 | 7 | 0.1 | 7 |
| AL_4 | USA-Alabama | AL_A | AL_A4 | 25 | 16 | 5 | 8 | 1.1 | 10 |
| AL_5 | USA-Alabama | AL_B | AL_B1 | 23 | 12 | 3 | 4 | 0.1 | 3 |
| AL_6 | USA-Alabama | AL_B | AL_B2 | 24 | 11 | 2 | 3 | 0.3 | 4 |
| AL_7 | USA-Alabama | AL_C | AL_C1 | 31 | 16 | 4 | 5 | 0.8 | 5 |
| AL_8 | USA-Alabama | AL_C | AL_C2 | 25 | 12 | 3 | 6 | 0.3 | 6 |
| AL_9 | USA-Alabama | AL_C | AL_C3 | 25 | 12 | 3 | 3 | 0.0 | 5 |
| MD_1 | USA-Maryland | MD_A | MD_A1 | 24 | 14 | 4 | 8 | 1.6 | 11 |
| MD_2 | USA-Maryland | MD_A | MD_A2 | 24 | 11 | 3 | 3 | 0.0 | 5 |
| MD_3 | USA-Maryland | MD_A | MD_A3 | 23 | 10 | 5 | 7 | 1.8 | 9 |
| MD_4 | USA-Maryland | MD_B | MD_B1 | 23 | 15 | 3 | 4 | 0.0 | 3 |
| MD_5 | USA-Maryland | MD_B | MD_B2 | 24 | 10 | 3 | 4 | 0.8 | 4 |
| MD_6 | USA-Maryland | MD_B | MD_B3 | 25 | 14 | 2 | 2 | 0.1 | 3 |
| MD_7 | USA-Maryland | MD_C | MD_C1 | 24 | 10 | 2 | 3 | 0.0 | 3 |
| MD_8 | USA-Maryland | MD_C | MD_C2 | 22 | 13 | 3 | 3 | 0.0 | 3 |
| MD_9 | USA-Maryland | MD_C | MD_C3 | 25 | 12 | 4 | 5 | 0.6 | 5 |
| AU_1 | Australia | AU_A | AU_A1 | 26 | 12 | 3 | 4 | 0.8 | 4 |
| AU_2 | Australia | AU_A | AU_A2 | 23 | 12 | 2 | 4 | 0.8 | 3 |
| AU_3 | Australia | AU_A | AU_A3 | 23 | 16 | 2 | 3 | 0.1 | 6 |
| AU_4 | Australia | AU_A | AU_A4 | 25 | 16 | 3 | 3 | 1.7 | 5 |
| AU_5 | Australia | AU_B | AU_B1 | 23 | 16 | 5 | 12 | 3.4 | 13 |
| AU_6 | Australia | AU_B | AU_B2 | 22 | 13 | 3 | 8 | 1.5 | 6 |
| AU_7 | Australia | AU_B | AU_B3 | 17 | 10 | 3 | 6 | 0.7 | 5 |
| AU_8 | Australia | AU_C | AU_C1 | 29 | 15 | 4 | 9 | 2.1 | 10 |
| AU_9 | Australia | AU_C | AU_C2 | 29 | 17 | 5 | 7 | 1.4 | 9 |
| AU_10 | Australia | AU_C | AU_C3 | 26 | 12 | 4 | 9 | 1.5 | 9 |
| IT_1 | Italy | IT_A | IT_A1 | 24 | 15 | 1 | 1 | 0.0 | 1 |
| IT_2 | Italy | IT_A | IT_A2 | 25 | 11 | 2 | 2 | 0.5 | 4 |
| IT_3 | Italy | IT_A | IT_A3 | 25 | 15 | 2 | 2 | 0.0 | 2 |
| IT_4 | Italy | IT_B | IT_B1 | 25 | 16 | 2 | 4 | 0.4 | 7 |

**Supplementary Table S4.** DNA microsatellite loci for the small hive beetle (*Aethina tumida*) that were used in this study. Marker names, primer names, forward and reverse sequences and size of the most common allele for each microsatellite marker are shown. Markers were created by Papach et al (2023b).

| **Marker** | **Primer name** | **Sequence 5'-3'** | **Size (bp)** |
| --- | --- | --- | --- |
| Atu-16a | Atu-16a-ACG8-F1 | CGCCGTTAACACGTAACACA | 118 |
|  | Atu-16a-ACG8-R1 | AGCGACCGCAGTTACTGAAT |  |
| Atu-18a | Atu-18a-CT10-F1 | AATACGCATGTTTGCCACTTC | 191 |
|  | Atu-18a-CT10-R1 | GGGTTTGGCCATCTGAAAAT |  |
| Atu-5a | Atu-5a-ATT17-F1 | AACACACAATGCAATGCAGTAA | 211 |
|  | Atu-5a-ATT17-R1 | ACTCTGCATGCCGATGTCTA |  |
| Atu-8 | Atu8-AAC9-F | ATGCCAATGCAACAACGTAA | 296 |
|  | Atu8-AAC9-R | TCCTGAACAGTTTCCTGACCT |  |
| Atu-9 | Atu9-CA13-R | TCACGACGGCAAGTTTAACA | 287 |
|  | Atu9-CA13-F | AAACCGTGGACCGTTAATTG |  |
| Atu-11 | Atu11-GT17-R | CGGCGTCTTGTGATGATTTA | 281 |
|  | Atu11-GT17-F | AAAAACAAACACGCGAATCC |  |
| Atu-14 | Atu14-CA17-F | CTTACACATTTCGGGCCATT | 217 |
|  | Atu14-CA17-R | CCCTTCTCTCAATAGATTGCTTT |  |
| Atu-17 | Atu17-GCT8-R | CATGATGGTTCGTGAGCAAC | 168 |
|  | Atu17-GCT8-F | TGGCCACTATAACAGCCAAT |  |
| Atu-10 | Atu10-GT10-R | TCACCACAACGTTGCAATAAT | 285 |
|  | Atu10-GT10-F | GGGCATCGGACAAAGAAATA |  |
| Atu-2 | Atu2-AG9-R | TGGAAAAGTGGTGGTGAGTG | 349 |
|  | Atu2-AG9-F | AAAATGTTGCTGGGTGTCGT |  |
